# Supplementary material for: First-line glaucoma monotherapy medication patterns in Finland during 1995–2019 based on a population-based study
Source: PLoS One. 2025 Jan 30;20(1):e0316835. doi: 10.1371/journal.pone.0316835 (PMC11781729; doi:10.1371/journal.pone.0316835)
Supplement: S1 Table — (DOCX) [file pone.0316835.s001.docx]

**S1 Table. Percentages of different glaucoma diagnoses among glaucoma patients with drug monotherapy as a first-line glaucoma therapy in the FinHealth 2017 Survey sample**

| **Verified glaucoma diagnosis** | **%** |
| --- | --- |
| Primary/chronic open-angle glaucoma | 41.2 |
| Exfoliative glaucoma | 16.8 |
| Normal-tension glaucoma | 10.1 |
| Unspecified glaucoma | 10.1 |
| Suspected glaucoma | 6.7 |
| Chronic angle-closure glaucoma | 5.0 |
| Glaucoma secondary to other disorder/factor | 5.0 |
| Unspecified open-angle glaucoma | 1.7 |
| Acute angle-closure glaucoma | 1.7 |
| Pigmentary glaucoma | 0.8 |
| Total | n = 119 |

Diagnosis data were based on Care Registers for Social Welfare and Health Care inpatient care data during 1996–2019 and specialized health care outpatient visit data during 2011–2019.
