# Supplementary material for: First-line glaucoma monotherapy medication patterns in Finland during 1995–2019 based on a population-based study
Source: PLoS One. 2025 Jan 30;20(1):e0316835. doi: 10.1371/journal.pone.0316835 (PMC11781729; doi:10.1371/journal.pone.0316835)

PLOS ONE

First-line glaucoma monotherapy medication patterns in Finland during 1995–2019 based on a population-based study

--Manuscript Draft--

**Manuscript Number:**	PONE-D-24-25991
**Article Type:**	Research Article
**Full Title:**	First-line glaucoma monotherapy medication patterns in Finland during 1995–2019 based on a population-based study
**Short Title:**	First-line glaucoma medication patterns in Finland during 1995–2019
**Corresponding Author:**	Petri K. M. Purola, Ph.D.<br>Tampere University<br>Tampere, FINLAND
**Keywords:**	beta-blocker; glaucoma drug; latanoprost; nationwide survey; prescription; prostaglandin; persistence; refill; register data; timolol
**Abstract:**	Background: The long-term patterns in first-line glaucoma medication are not well established. Exploring these in longitudinal and population-based settings would provide information for the healthcare systems to plan glaucoma care accordingly.<br>Objective: To evaluate patterns in first-line glaucoma monotherapy in Finland during 1995–2019 based on nationwide survey and register data.<br>Methods: A population-based cohort study with 25 years of total follow-up. The cohort (n = 9288) is a random sample drawn for the nationwide health examination survey FinHealth 2017 which represents the Finnish population aged 30 years or older in 2017. Glaucoma patients were selected from the survey participants based on linked register data that included prescriptions and special reimbursements for glaucoma medication. The patterns, length of use, age at initiation, and persistence of first-line glaucoma drug monotherapies were observed during 1995–2019.<br>Results: A total of 141 glaucoma patients with glaucoma drug monotherapy as a first-line glaucoma therapy were identified from the sample. The proportion of patients continuing with their first-line monotherapy was 64% after 1 year, 37% after 3 years, and 21% after 5 years of follow-up. During the 25 years there was a shift from beta-blockers to prostaglandin analogues as the prominent first-line glaucoma drug class. The length of use was statistically significantly longer for prostaglandin analogues compared with beta-blockers among patients continuing with their first-line monotherapy after 5 years of follow-up. The non-persistence rate was 38% of all patients during their first-line monotherapy. Timolol fixed-combinations were the most common second-line glaucoma therapy with a share of 39% after 5 years of follow-up.<br>Conclusions: During the 25-year follow-up a shift from beta-blockers to prostaglandin analogues has occurred and long initial therapies of over 5 years have become more common. However, the decline in the continuation of the initial therapy still occurs early with 1 out of 3 patients continuing after 3 years. This decline together with the consistent problem of non-persistence remain a clinical challenge in topical drug therapy of glaucoma.
**Order of Authors:**	Petri K. M. Purola, Ph.D.
	Seppo V. P. Koskinen, Research Professor
	Hannu M. T. Uusitalo, Professor Emeritus
**Additional Information:**	
**Question**	**Response**
**Financial Disclosure**<br><br>Enter a financial disclosure statement that describes the sources of funding for the work included in this submission. Review the submission guidelines for detailed	Yes

Powered by Editorial Manager® and ProduXion Manager® from Aries Systems Corporation

requirements. View published research articles from *PLOS ONE* for specific examples.<br><br>This statement is required for submission and **will appear in the published article** if the submission is accepted. Please make sure it is accurate.<br><br>**Funded studies**<br>Enter a statement with the following details:<br>• Initials of the authors who received each award<br>• Grant numbers awarded to each author<br>• The full name of each funder<br>• URL of each funder website<br>• Did the sponsors or funders play any role in the study design, data collection and analysis, decision to publish, or preparation of the manuscript?<br><br>Did you receive funding for this work?	
Please add funding details.<br>as follow-up to "**Financial Disclosure**<br><br>Enter a financial disclosure statement that describes the sources of funding for the work included in this submission. Review the submission guidelines for detailed requirements. View published research articles from *PLOS ONE* for specific examples.<br><br>This statement is required for submission and **will appear in the published article** if the submission is accepted. Please make sure it is accurate.	PKMP has received research grants from Tampereen seudun Näkövammaisten tukisäätiö s.r, Tampere, Finland (https://www.tsnvtukisaatio.fi); Elsemay Björn Fund, Helsinki, Finland (URL not available); Finnish Federation of the Visually Impaired, Helsinki, Finland (https://www.nkl.fi); Emil Aaltosen Säätiö, Tampere, Finland (https://emilaaltonen.fi); and Juho Vainion Säätiö, Helsinki, Finland (www.juhovainionsaatio.fi). The funders had no role in study design, data collection and analysis, decision to publish, or preparation of the manuscript.

**Funded studies**<br>Enter a statement with the following details:<br>• Initials of the authors who received each award<br>• Grant numbers awarded to each author<br>• The full name of each funder<br>• URL of each funder website<br>• Did the sponsors or funders play any role in the study design, data collection and analysis, decision to publish, or preparation of the manuscript?<br><br>Did you receive funding for this work?"	
Please select the country of your main research funder (please select carefully as in some cases this is used in fee calculation).<br>as follow-up to "**Financial Disclosure**<br><br>Enter a financial disclosure statement that describes the sources of funding for the work included in this submission. Review the submission guidelines for detailed requirements. View published research articles from *PLOS ONE* for specific examples.<br><br>This statement is required for submission and **will appear in the published article** if the submission is accepted. Please make sure it is accurate.<br><br>**Funded studies**<br>Enter a statement with the following details:<br>• Initials of the authors who received each award<br>• Grant numbers awarded to each author<br>• The full name of each funder<br>• URL of each funder website<br>• Did the sponsors or funders play any role in the study design, data collection and analysis, decision to publish, or preparation of the manuscript?<br><br>Did you receive funding for this work?"	FINLAND - FI
**Competing Interests**	The authors have declared that no competing interests exist.

Use the instructions below to enter a competing interest statement for this submission. On behalf of all authors, disclose any competing interests that could be perceived to bias this work—acknowledging all financial support and any other relevant financial or non-financial competing interests.<br><br>This statement is required for submission and **will appear in the published article** if the submission is accepted. Please make sure it is accurate and that any funding sources listed in your Funding Information later in the submission form are also declared in your Financial Disclosure statement.<br><br>View published research articles from *PLOS ONE* for specific examples.<br><br>**NO authors have competing interests**<br><br>Enter: *The authors have declared that no competing interests exist.*<br><br>**Authors with competing interests**<br><br>Enter competing interest details beginning with this statement:<br><br>*I have read the journal's policy and the authors of this manuscript have the following competing interests: [insert competing interests here]*<br><br>* typeset	
**Ethics Statement**<br><br>Enter an ethics statement for this submission. This statement is required if the study involved:<br><br>• Human participants<br>• Human specimens or tissue<br>• Vertebrate animals or cephalopods<br>• Vertebrate embryos or tissues	The FinHealth 2017 Survey was approved by the Coordinating Ethics Committee at the Hospital District of Helsinki and Uusimaa with approval number 37/13/02/00/2016 (please see attachments). Written informed consent was obtained from all participants.

Powered by Editorial Manager® and ProduXion Manager® from Aries Systems Corporation

• Field research<br><br>Write "N/A" if the submission does not require an ethics statement.<br><br>General guidance is provided below. Consult the submission guidelines for detailed instructions. **Make sure that all information entered here is included in the Methods section of the manuscript.**	

**Format for specific study types**<br><br>**Human Subject Research (involving human participants and/or tissue)**<br>• Give the name of the institutional review board or ethics committee that approved the study<br>• Include the approval number and/or a statement indicating approval of this research<br>• Indicate the form of consent obtained (written/oral) or the reason that consent was not obtained (e.g. the data were analyzed anonymously)<br><br>**Animal Research (involving vertebrate animals, embryos or tissues)**<br>• Provide the name of the Institutional Animal Care and Use Committee (IACUC) or other relevant ethics board that reviewed the study protocol, and indicate whether they approved this research or granted a formal waiver of ethical approval<br>• Include an approval number if one was obtained<br>• If the study involved *non-human primates*, add *additional details* about animal welfare and steps taken to ameliorate suffering<br>• If anesthesia, euthanasia, or any kind of animal sacrifice is part of the study, include briefly which substances and/or methods were applied<br><br>**Field Research**<br><br>Include the following details if this study involves the collection of plant, animal, or other materials from a natural setting:<br>• Field permit number<br>• Name of the institution or relevant body that granted permission	
**Data Availability**<br><br>Authors are required to make all data underlying the findings described fully available, without restriction, and from the time of publication. PLOS allows rare exceptions to address legal and ethical concerns. See the PLOS Data Policy and FAQ for detailed information.	No - some restrictions will apply

Powered by Editorial Manager® and ProduXion Manager® from Aries Systems Corporation

A Data Availability Statement describing where the data can be found is required at submission. Your answers to this question constitute the Data Availability Statement and **will be published in the article**, if accepted.<br><br>**Important:** Stating 'data available on request from the author' is not sufficient. If your data are only available upon request, select 'No' for the first question and explain your exceptional situation in the text box.<br><br>Do the authors confirm that all data underlying the findings described in their manuscript are fully available without restriction?	
**Describe where the data may be found in full sentences. If you are copying our sample text, replace any instances of XXX with the appropriate details.**<br><br>• If the data are **held or will be held in a public repository**, include URLs, accession numbers or DOIs. If this information will only be available after acceptance, indicate this by ticking the box below. For example: *All XXX files are available from the XXX database (accession number(s) XXX, XXX.).*<br>• If the data are all contained **within the manuscript and/or Supporting Information files**, enter the following: *All relevant data are within the manuscript and its Supporting Information files.*<br>• If neither of these applies but you are able to provide **details of access elsewhere**, with or without limitations, please do so. For example:<br><br>*Data cannot be shared publicly because of [XXX]. Data are available from the XXX Institutional Data Access / Ethics Committee (contact via XXX) for researchers who meet the criteria for access to confidential data.*<br><br>*The data underlying the results presented in the study are available from (include the name of the third party*	The Ethics Committee has stated that under applicable law, the study data cannot be made publicly available or stored in public repositories because individual level data contain sensitive health information. Anonymized data can be requested for a study that has been approved by a local ethics committee and meet the criteria for access to confidential data. The permission to access the data can be applied through the Finnish Institute for Health and Welfare (thl.fi/finhealth).

and contact information or URL).<br>• This text is appropriate if the data are owned by a third party and authors do not have permission to share the data.<br><br>* typeset	
Additional data availability information:	

Powered by Editorial Manager® and ProduXion Manager® from Aries Systems Corporation

Manuscript Click here to access/download;Manuscript;Manuscript.docx

First-line glaucoma monotherapy medication patterns in Finland during 1995–2019 based on a population-based study

*Petri K.M. Purola[1,2], Seppo V.P. Koskinen[3], Hannu M.T. Uusitalo[1,2,4]

[1]Department of Ophthalmology, Faculty of Medicine and Health Technology, Tampere University, Tampere, Finland

[2]Finnish Register of Visual Impairment, Finnish Federation of the Visually Impaired, Helsinki, Finland

[3]Department of Public Health and Welfare, Finnish Institute for Health and Welfare, Helsinki, Finland

[4]Tays Eye Center, Tampere University Hospital, Tampere, Finland

* Corresponding author

E-Mail: petri.purola@tuni.fi (PKMP)

Short title: First-line glaucoma medication patterns in Finland during 1995–2019

Abstract

Background: The long-term patterns in first-line glaucoma medication are not well established. Exploring these in longitudinal and population-based settings would provide information for the healthcare systems to plan glaucoma care accordingly.

Objective: To evaluate patterns in first-line glaucoma monotherapy in Finland during 1995–2019 based on nationwide survey and register data.

Methods: A population-based cohort study with 25 years of total follow-up. The cohort (*n* = 9288) is a random sample drawn for the nationwide health examination survey FinHealth 2017 which represents the Finnish population aged 30 years or older in 2017. Glaucoma patients were selected from the survey participants based on linked register data that included prescriptions and special reimbursements for glaucoma medication. The patterns, length of use, age at initiation, and persistence of first-line glaucoma drug monotherapies were observed during 1995–2019.

Results: A total of 141 glaucoma patients with glaucoma drug monotherapy as a first-line glaucoma therapy were identified from the sample. The proportion of patients continuing with their first-line monotherapy was 64% after 1 year, 37% after 3 years, and 21% after 5 years of follow-up. During the 25 years there was a shift from beta-blockers to prostaglandin analogues as the prominent first-line glaucoma drug class. The length of use was statistically significantly longer for prostaglandin analogues compared with beta-blockers among patients continuing with their first-line monotherapy after 5 years of follow-up. The non-persistence rate was 38% of all patients during their first-line monotherapy. Timolol fixed-combinations were the most common second-line glaucoma therapy with a share of 39% after 5 years of follow-up.

Conclusions: During the 25-year follow-up a shift from beta-blockers to prostaglandin analogues has occurred and long initial therapies of over 5 years have become more

common. However, the decline in the continuation of the initial therapy still occurs early with 1 out of 3 patients continuing after 3 years. This decline together with the consistent problem of non-persistence remain a clinical challenge in topical drug therapy of glaucoma.

Introduction

Glaucoma is a collection of optic neuropathies that can lead to irreversible blindness if left untreated. Elevated intraocular pressure (IOP) is considered the principal risk factor for glaucoma [1]. Therefore, the current treatment of glaucoma aims to prevent the progression of the disease and consequent vision loss by lowering the IOP via medication, laser treatments, or surgery [1,2].

The treatment of glaucoma is usually initiated with topical antihypertensive and recommended to be initiated as a monotherapy but can be switched to another monotherapy or a combination drug therapy with two or more IOP-lowering agents in the case the current therapy is deemed insufficient [3,4]. Alternatively, laser treatment or surgery can be offered as a first-line glaucoma therapy in poorly adherent patients, in patients with severe disease, and in patients with difficulties in administering topical medication.

Glaucoma treatment requires lifelong adherence and frequent follow-up. The adherence to the recommended medication among glaucoma patients is a key challenge for treatment success in glaucoma. Numerous studies have demonstrated that low adherence and persistence with topical and systemic glaucoma treatment is common [5–7], which can manifest as progression of vision loss and eventual blindness [8].

However, the long-term patterns in the first-line glaucoma medication are not well established. Previous studies on the subject have been limited to short, less than 10-year follow-up periods [9–13] and samples that are not representative at the population level [12,14]. Exploring these patterns in longitudinal and population-based settings with comprehensive follow-up periods would provide highly needed information for the healthcare

systems to plan glaucoma care accordingly. Therefore, in the present study our aim was to analyze the patterns in the first-line glaucoma medication during a total follow-up period of 25 years based on a population-representative cohort of Finnish adults from a nationwide health examination survey complemented with national health register data on glaucoma and glaucoma medication.

Materials and methods

Study design and data

This study is based on FinHealth 2017, a cross-sectional nationwide health examination survey conducted in 2017 by the Finnish Institute for Health and Welfare (THL) [15]. The goal of the survey was to collect comprehensive, up-to-date information on health, functional capacity, and welfare of Finnish adults. The survey included comprehensive self-reported assessments as well as a health examination conducted at a nearby screening center.

The survey included a nationally representative sample of 10,247 Finnish adults aged 18 years or older with a participation rate of 68.8% for participating at any phase of data collection. The details of the design and sampling of the survey have been described previously [16]. To summarize, the sample was drawn by a two-stage stratified cluster sampling, and persons aged 80 or older were oversampled by doubling the sampling fraction. The sample weights were calibrated by post-stratification defined by age, sex, region, and native language to account for non-response and missing data. Separate weights were applied for the survey to produce results representing the Finnish adult population in 2017. For the present study we selected the 9,288 participants aged 30 years or older as the study cohort.

The survey sample was linked to the Social Insurance Institution of Finland (KELA) registers to obtain data on the special reimbursement for glaucoma medication during 1972–2019 and

the number of glaucoma medication prescriptions during 1995–2019. Data on glaucoma-related diagnoses and operations were obtained from the Care Registers for Social Welfare and Health Care based on inpatient care (HILMO) during 1996–2019 as well as glaucoma-related diagnoses based on specialized health care outpatient visits (AvoHILMO) during 2011–2019. The HILMO and AvoHILMO data are collected automatically from healthcare service providers' patient management systems and delivered to THL.

Glaucoma patient selection

We classified survey participants into glaucoma patients according to the following register-based conditions: 1) entitlement to special reimbursement for glaucoma medication based on KELA data during 1972–2019; or 2) glaucoma diagnosis (International Classification of Diseases diagnosis codes H40 and H40.1–H40.9 for version 10) based on HILMO data during 1996–2019 and AvoHILMO data during 2011–2019; or 3) glaucoma-related eye operation (NOMESCO Classification for Surgical Procedures CHB50, CHD00, CHD05, CHD15, CHD50, CHD60, CHD65, CHD99, and CHF05) based on HILMO data during 1996–2019.

Glaucoma medication data

In Finland, glaucoma medication is distributed by pharmacies every 3 months, and the data on the number and type of used glaucoma medications during these 3-month periods were available for all study participants. Glaucoma drugs were classified based on glaucoma medication prescriptions and refills with ATC code S01E* obtained from KELA during 1995–2019. To allow the scrutinization of first-line glaucoma drugs we included only those with glaucoma medication initiated with drug monotherapy during 1995–2019.

The age at initiation of therapy was based on the date of the first prescription. The length of use was calculated as the time between the date of the first prescription of the first drug and 1) the date of the first glaucoma-related eye operation; or 2) the date of the first prescription

of the second drug if preceding a possible glaucoma-related eye operation; or 3) the last refill of the first drug if it was not switched during the follow-up.

The analyses were based on both the drug class and its type. The classes (and types) included beta-blockers (betaxolol, timolol), alpha-2 agonists (brimonidine), prostaglandin analogues (bimatoprost, latanoprost, tafluprost, travoprost), carbonic anhydrase inhibitors (brinzolamide, dorzolamide), and other (apraclonidine, pilocarpine).

Statistical analyses

All analyses were carried out using R software (v. 4.3.1, R Core Team, R Foundation for Statistical Computing, Austria). The sampling design of the survey was accounted for using Survey package 4.2-1 for R [17] and weighting scheme calculated by THL. The results were expressed as means, standard deviations (SDs), and 95% confidence intervals (CIs). A Kaplan–Meier estimator was created and tested with log-rank and Gehan–Wilcoxon test using package survival 3.5-7 [18,19]. A Sankey diagram was drawn using package PantaRhei 0.1.2 [20]. The distribution of the analyzed data was skewed according to Shapiro–Wilk normality test. Therefore, Mann–Whitney U test was used for between-group comparisons and Kruskal–Wallis test to compare multiple groups adjusted with Dunn–Bonferroni correction using function DunnTest from package DescTools 0.99.50 [21]. There were no outliers. All p-values were two-sided and were considered statistically significant when less than 0.05.

Ethics approval and informed consent

The current study was conducted in line with the tenets of the Helsinki Declaration. All procedures in the FinHealth 2017 Survey involving human participants were performed in accordance with the ethical standards of the institutional and national research committees, and with the 1964 Helsinki declaration and its later amendments or comparable ethical standards. The FinHealth 2017 Survey was approved by the Coordinating Ethics Committee

at the Hospital District of Helsinki and Uusimaa. All participants received an information letter regarding the study beforehand. Written informed consent was obtained from all participants. The ethical approval process details have been reported in a previous publication [16].

Data availability

The Ethics Committee has stated that under applicable law, the study data cannot be made publicly available or stored in public repositories because individual level data contain sensitive health information. Anonymized data can be requested for a study that has been approved by a local ethics committee and meet the criteria for access to confidential data. The permission to access the data can be applied through THL (thl.fi/finhealth).

Results

Out of the total 9,288 participants aged 30 years or older in the FinHealth 2017 Survey, 6,425 (69.2%) had partaken in at least one part of the survey. The selection process of the analyzed participants from this eligible sample is illustrated in Fig 1. A total of 203 glaucoma patients treated with medication were observed in the sample, of which 62 were excluded: 43 (21%, 43/203) started with multitherapy as a first-line glaucoma therapy, 4 had existing multitherapy since start of the follow-up in 1995, 12 were treated with laser therapy or surgery as a first-line glaucoma therapy, and 3 had only a single glaucoma medication prescription during the follow-up. A total of 141 (69%, 141/203) glaucoma patients with drug monotherapy as a first-line glaucoma therapy were selected for the analyses. During the 25 years of total follow-up 59% (n = 83) of the analyzed participants switched to another drug, 18% (n = 26) had no change in their initial therapy during the follow-up, 16% (n = 23) had a second drug added alongside the first-line drug, and 6% (n = 9) were treated with laser therapy or cataract surgery as a second-line therapy.

Fig 1. Flowchart of the selection of glaucoma patients treated with first-line monotherapy in the FinHealth 2017 Survey sample representing the Finnish adult population aged 30 years or older. SD = standard deviation.

The distribution of first-line glaucoma drug classes and types in the survey sample is shown in Table 1. The proportion of patients continuing with their first-line glaucoma monotherapy after 1, 3, and 5 years of follow-up is shown in Table 2. The proportion of patients continuing with their initial monotherapy among all first-line glaucoma drugs was 64% (90/141) after 1 year, 37% (52/141) after 3 years, and 21% (29/141) after 5 years of follow-up. Prostaglandin analogues were the most common first-line glaucoma drug class with a share of 70% (98/141) followed by beta-blockers with 23% (33/141). Latanoprost was the most common first-line glaucoma drug type with a share of 38% followed by timolol with 20%, tafluprost with 13%, travoprost with 12%, and bimatoprost with 6%.

Table 1. Number and proportion of glaucoma patients in the FinHealth 2017 Survey sample representing the Finnish adult population aged 30 years or older according to their first-line glaucoma monotherapy drug class and type during the follow-up between 1995 and 2019

**First-line glaucoma drug class and type**	**n and %**
Prostaglandin analogue	98 (70%)
Latanoprost	54 (38%)
Tafluprost	19 (13%)
Travoprost	17 (12%)
Bimatoprost	8 (6%)
Beta-blocker	33 (23%)
Timolol	28 (20%)
Betaxolol	5 (4%)
Carbonic anhydrase inhibitor	4 (3%)
Brinzolamide	3 (2%)
Dorzolamide	1 (1%)
Alpha-2 agonist	3 (2%)
Brimonidine	3 (2%)
Other	3 (2%)
Pilocarpine	2 (1%)

Apraclonidine	1 (1%)
Total	141 (100%)

Table 2. Number and proportion of glaucoma patients continuing with their first-line therapy after 1, 3, and 5 years of follow-up in the FinHealth 2017 Survey sample representing the Finnish adult population aged 30 years or older according to their first-line glaucoma monotherapy drug class and type during the follow-up between 1995 and 2019

**First-line glaucoma drug class and type**	**n at the start**	**n and % continuing after 1 year**	**n and % continuing after 3 years**	**n and % continuing after 5 years**
Prostaglandin analogue	98	61 (62%)	34 (35%)	19 (19%)
Latanoprost	54	36 (67%)	19 (35%)	13 (24%)
Tafluprost	19	9 (47%)	5 (26%)	3 (16%)
Travoprost	17	11 (65%)	8 (47%)	2 (12%)
Bimatoprost	8	5 (63%)	2 (25%)	1 (13%)
Beta-blocker	33	24 (73%)	15 (46%)	9 (27%)
Timolol	28	22 (79%)	13 (46%)	7 (25%)
Betaxolol	5	2 (40%)	2 (40%)	2 (40%)
Carbonic anhydrase inhibitor	4	2 (50%)	2 (50%)	0 (0%)
Brinzolamide	3	2 (67%)	2 (67%)	0 (0%)
Dorzolamide	1	0 (0%)	0 (0%)	0 (0%)
Alpha-2 agonist	3	1 (33%)	0 (0%)	0 (0%)
Brimonidine	3	1 (33%)	0 (0%)	0 (0%)
Other	3	2 (67%)	1 (33%)	1 (33%)
Pilocarpine	2	1 (50%)	1 (50%)	1 (50%)
Apraclonidine	1	1 (100%)	0 (0%)	0 (0%)
Total	141	90 (64%)	52 (37%)	29 (21%)

The non-persistence regarding all first-line glaucoma monotherapies as well as prostaglandin analogues and beta-blockers in specific is shown in Table 3 and Fig 2. Overall, 62% of all patients did not have significant delays (refills delayed < 1 months) between their refills during their first-line monotherapy, while 38% had at least 1 refill delayed ≥ 1 months and 20% at least 1 refill delayed ≥ 3 months. The persistence did not differ noticeably between prostaglandin analogues and beta-blockers. During the 25-year follow-up the proportion of delays of 1 ≥ months decreased while the proportion of delays of

3 ≥ months increased. The persistence for the other drug classes and types in specific was not determined due to low number of participants (*n* ≤ 5).

Table 3. Non-persistence regarding all first-line glaucoma monotherapies and the two most common glaucoma drug classes during the follow-up between 1995 and 2019 in the FinHealth 2017 Survey sample representing the Finnish adult population aged 30 years or older

	**n and %**						
**First-line glaucoma drug class**	**Patients treated with first-line monotherapy**	**Patients with at least 1 refill delayed ≥ 1 months**	**Patients with at least 1 refill delayed ≥ 3 months**		**Refills**	**Refills delayed ≥ 1 months**	**Refills delayed ≥ 3 months**
Prostaglandin analogue	98	37 (38%)	20 (20%)		965	117 (12%)	44 (5%)
Beta-blocker	33	14 (42%)	6 (18%)		304	50 (17%)	6 (2%)
All	141	54 (38%)	28 (20%)		1381	172 (12%)	52 (4%)

Other first-line glaucoma drug classes are not listed due to low number of participants.

Fig 2. Non-persistence in first-line glaucoma monotherapy during the follow-up between 1995 and 2019 in the FinHealth 2017 Survey sample representing the Finnish adult population aged 30 years or older. Numbers were smoothed using a 3-year central moving average starting from 1997 with a 2-year wash-out period included at the start of the follow-up.

The time trends in the two most common first-line glaucoma drugs classes, first-line (selective) laser trabeculoplasty, and the most common second-line therapy of the cohort, timolol fixed-combinations, during 1995–2019 are presented in Fig 3. Between 1997 and 2002 beta-blockers were the most common first-line glaucoma drug class. The use of prostaglandin analogues increased during 1999–2002, and from 2003 onwards they have remained as the most prevalent first-line glaucoma drug class.

Fig 3. Time trends in glaucoma treatment during the follow-up between 1995 and 2019 in the FinHealth 2017 Survey sample representing the Finnish adult population aged 30 years or older. Numbers were smoothed using a 3-year central moving average starting from 1997 with a 2-year wash-out period included at the start of the follow-up.

The length of use of the two most common first-line glaucoma drug classes and the five most common drug types during 1995–2019 is illustrated in Fig 4 as survival curves. No statistically significant differences in the length of use were detected between the two glaucoma drug classes and the five glaucoma drug types during the total follow-up time. However, after 5 years of follow-up the length of use was statistically significantly longer for prostaglandin analogues compared with beta-blockers according to the log-rank test ($p = 0.02$). After 7 years of follow-up this difference was significant also according to the Gehan–Wilcoxon test ($p = 0.02$). The length of use for the other drug classes and types were not determined due to low number of participants ($n \leq 5$).

Fig 4. Plot of Kaplan–Meier estimator on the length of use of the two most common first-line glaucoma monotherapy drug classes and the five most common drug types during the follow-up between 1995 and 2019 in the FinHealth 2017 Survey sample representing the Finnish adult population aged 30 years or older. Participants without switch to second-line therapy during the follow-up were considered right-censored with their endpoints shown as vertical lines. After 5 years of follow-up there was a significant difference in the length of use between prostaglandin analogues and beta-blockers according to the log-rank test ($p = 0.02$), and after 7 years the difference was significant also according to the Gehan–Wilcoxon test ($p = 0.02$).

The mean age at initiation of therapy of the two most common first-line glaucoma drug classes and the five most common drug types during 1995–2019 is illustrated in Fig 5. The mean age at initiation was significantly ($p = 0.002$) lower for beta-blockers with a mean of 59.9 years (95% CI 56.3–63.4) in comparison to prostaglandin analogues with a mean of 66.9 years (95% CI 64.6–69.1). In relation to this, post hoc pairwise tests determined timolol with a mean of 59.6 years (95% CI 56.0–63.1) being associated with the lowest age at initiation in comparison to those of the four other common drugs ($p = 0.005$), which were

65.5 years (95% CI 62.5–68.6) for latanoprost, 69.4 years (95% CI 65.0–73.8) for tafluprost, 68.1 years (95% CI 63.7–72.6) for travoprost, and 66.6 years (95% CI 61.3–71.8) for bimatoprost. This difference is likely explained by the fact that the use of timolol was particularly common during 1998–2002 when the 2017 cohort was 15–19 years younger (Fig 3). The mean age at initiation for the other drug classes and types were not determined due to low number of subjects ($n \leq 5$).

Fig 5. Mean age at initiation of therapy of the two most common first-line glaucoma monotherapy drug classes and the five most common drug types during the follow-up between 1995 and 2019 in the FinHealth 2017 Survey sample representing the Finnish adult population aged 30 years or older

The patterns in switching from the five most common first-line glaucoma drug types to second-line therapies during 1, 3, and 5 years of follow-up and during total follow-up in 1995–2019 are illustrated by Sankey diagrams in Fig 6. The proportions of patients continuing with their first-line glaucoma drug after 1, 3, and 5 years of follow-up and total follow-up were 72%, 41%, 33%, and 24% for latanoprost; 81%, 50%, 29%, and 0% for timolol; 53%, 38%, 38%, and 42% for tafluprost; 69%, 53%, 14%, and 12% for travoprost; and 63%, 29%, 17%, and 25% for bimatoprost, respectively. Timolol fixed-combinations were the most common second-line therapy with a share of 17% (20/118), 32% (34/107), and 39% (36/92) after 1, 3, and 5 years of follow-up, respectively, and its share was 44% (55/126) of all second-line therapies used during the total follow-up.

Fig 6. Sankey diagram illustrating the number of glaucoma patients treated with the five most common first-line glaucoma drugs and the number of patients who either had a switch to second-line therapy or who had no change to their therapy during (A) 1 year of follow-up, (B) 3 years of follow-up, (C) 5 years of follow-up, and (D) total follow-up during 1995–2019 in the FinHealth 2017 Survey sample representing the Finnish adult population aged 30 years or older. Patients with shorter follow-up per respective follow-up period (A–C) were excluded. All patients were included in the total

follow-up (D). The percentages of the most common switches or continuations in relation to the first-line glaucoma drugs are also shown.

Discussion

Due to many individual-based factors that need to be accounted for in glaucoma therapy, glaucoma patients usually undergo many treatment modifications. The knowledge of these is limited due to lack of population-based data and extensive follow-up periods. A detailed investigation of glaucoma medication patterns would provide valuable information on patient behavior and used glaucoma therapies for the healthcare systems to adjust and improve glaucoma care, particularly as the demand for glaucoma care is expected to increase due to the ageing of the populations and the increasing life expectancy. To fill this gap in knowledge, we investigated the patterns of first-line glaucoma drug monotherapies in Finland during a 25-year follow-up period based on a population-based cohort. This is to our knowledge the longest follow-up which has been used in determining these patterns. By providing time trends in glaucoma management during the recent decades, the current study can aid in planning how to best meet the increased glaucoma care needs in the future.

Our main finding is that the decline in the continuation of the initial monotherapy occurs early in the therapy journey: the proportion of patients continuing with their initial glaucoma drug monotherapy had declined to 64% after 1 year and it further declined to 21% after 5 years of follow-up. Non-persistence is still a common and consistent problem with 38% of patients having significant delays in refills during their first-line glaucoma monotherapy. However, the length of use of the first-line glaucoma monotherapy of over 5 years has prolonged during the shift from beta-blockers to prostaglandin analogues as the prominent first-line glaucoma drug in the 2000s. Timolol fixed-combinations were the most prominent second-line glaucoma therapy with a share of 39% after 5 years of follow-up.

In our study, 64%, 37% and 21% of patients continued with their first-line glaucoma monotherapy after 1, 3, and 5 years of follow-up, respectively. This is noticeably lower than in a previous healthcare insurance claims database study conducted in the US during 2010–2014, in which 56% of newly diagnosed glaucoma patients continued with their initial drug monotherapy during the first 4 years of treatment [13]. On the other hand, based on a systematic literature review, 40% continued with their initial therapy after 1 year from therapy start without discontinuation or change of therapy [7], which is lower than in our study; however, their percentage is based on a mean from 12 studies, in which the percentage individually ranged from 14% to 67% [7].

Although no significant difference in the length of use was observed between glaucoma drug classes and types during the first 5 years of treatment in our study, among those continuing with their first-line treatment after 5 years of follow-up prostaglandin analogues were associated with a significantly longer length of use in comparison to beta-blockers. In line with our results, Goldshtein et al. reported a noticeably longer median time till discontinuation or switching among first-line prostaglandins (3.6 years) compared with other drug classes based a population-based study conducted in Israel in 2003–2012 [11]. However, they observed a median time of 1.8 years for first-line beta-blockers, whereas in our study the mean time of continuation was 3.5 years. This could be explained by the follow-up period in our study starting earlier, in the 1990s when beta-blockers were more commonly used as a first-line glaucoma monotherapy. Interestingly, Diestelhorst et al. reported that as early as in 1996–1998 patients who initially received latanoprost stayed on therapy more than twice as long as did those treated first with a beta-blocker during a 2-year follow-up based on a multicenter medical chart review study conducted in multiple countries [9].

Prostaglandin analogues, latanoprost in particular, were the most prominent first-line glaucoma monotherapy drug during the total 25-year follow-up in our study: the share of prostaglandin analogues was 70% of all glaucoma drug classes, and the share of

latanoprost was 38% of all glaucoma drug types. They were followed by beta-blockers and timolol with respective shares of 23% and 20%. After the introduction of latanoprost in Finland in 1997 its use increased quickly, and from 2003 onwards prostaglandin analogues have remained as the most used first-line glaucoma drug class in Finland. In similar, prostaglandins have also been reported in many other studies as the prominent prescribed medical therapy in the 2000s followed by beta-blockers [12,22,23]. Interestingly, based on a population-based study conducted in Israel in 2003–2012, the most common first-line drug class for glaucoma were beta-blockers with a share of 45% followed by prostaglandins with 27% [11].

The non-persistence regarding first-line glaucoma monotherapies can be considered high in our study: 38% of all patients had one or more over 1-month delays between 3-month refills during their initial therapy. The rate of non-persistence has been reported higher in previous studies [24–27], although the results may not be directly compared due to differences in the length of delay and follow-up as well as the inclusion of other than first-line monotherapies. Furthermore, in Finland glaucoma medication is reimbursed 100% by KELA, which evidently is improving the persistence rate. The persistence was similar between prostaglandin analogues and beta-blockers in our study, whereas in previous studies prostaglandin analogues have been reported having higher persistence rates than other drug classes [9,24,25]. Interestingly, in our study, the proportion of all delayed refills declined during the 25-year follow-up while the proportion of the most severe delays somewhat increased, potentially reflecting the ageing of the study cohort.

In our study, approximately 21% of glaucoma patients treated with medication had their therapy initiated with multiple antiglaucoma agents. This figure is in line with a population-based study conducted in Israel in 2003–2012, in which 22% of glaucoma patients treated with medication had either a combination drug or several separate drugs with multitherapy as a first-line glaucoma therapy [11]. This behavior is contradictory to the glaucoma guideline [28], and the reason for it is not known.

Timolol fixed-combinations were the most common second-line therapy according to our data with its share increasing from 17% to 39% after 1 and 5 years of follow-up, respectively, and its total share was 44% of all second-line therapies. Its use as a second-line therapy increased noticeably between 1995 and 2019. In relation to our results, based on a population-based data from 2003 to 2017 in Australia the use of fixed-combination medications showed a 9.2-fold increase between the two time points [22].

A 4.6-fold increase in all cases of laser trabeculoplasty was observed between 2003 and 2017 in Australia [22]. No similar trend was observed in our study. Although selective laser trabeculoplasty has been suggested as a safe and effective first-line alternative to glaucoma treatment, is not routinely offered as first-line treatment [29], which probably explains the absence of visible trends in laser operations in our study. Furthermore, our data has limited coverage of the selective laser trabeculoplasty in private sector, which is likely to further reduce the number of laser operations in our dataset.

The main strength of our study was the use of data from a nationwide survey linked to comprehensive register data follow-up for 25 years. The survey sample represents the Finnish adult population in 2017, and the survey addressed public health issues more comprehensively than national health surveys do on average. In addition, the study population did not consist of specific patient groups collected from health-care units allowing for better generalization of the results. The participation rate in the survey was relatively high and was further corrected by applying the weights. The analyses were conducted on the largest possible number of participants. Details of prescription were available for each purchase allowing the determination of the precise expected refill date.

Our study also has limitations. The cohort was selected at a single time point, and as such, the distribution of initial glaucoma therapies per year does not represent the distribution at population level each year. Similarly, comparing the age at initiation of therapy between glaucoma drug classes and types is biased at the early years of the follow-up due to the

younger age of the cohort. As such, the significantly younger age at initiation of timolol monotherapies should be interpreted with caution. The survey included predominantly participants of Finnish origin; therefore, the results may not be directly applicable to other countries. In addition, interpretation of the differences between our results and those of previous studies can be rather difficult due to the heterogeneity of methods for measuring continuation and persistence and the differences in patient selection criteria.

In conclusion, the decline in the continuation of the initial glaucoma monotherapy occurs early in the therapy journey, although the length of use has prolonged among glaucoma patients who have continued with first-line prostaglandin analogues for more than 5 years. The non-persistence to first-line glaucoma monotherapies remains a significant issue. There is a need for education and tailoring of the therapeutic scheme particularly for glaucoma patients at the start of the therapy journey and monitoring the treatment scheme of all glaucoma patients during the follow-up.

References

1. Weinreb RN, Aung T, Medeiros FA. The Pathophysiology and Treatment of Glaucoma: A Review. JAMA. 2014;311: 1901–1911. doi:10.1001/jama.2014.3192

2. Heijl A, Leske MC, Bengtsson B, Hyman L, Bengtsson B, Hussein M, et al. Reduction of Intraocular Pressure and Glaucoma Progression: Results From the Early Manifest Glaucoma Trial. Arch Ophthalmol. 2002;120: 1268–1279. doi:10.1001/archopht.120.10.1268

3. Parkkari M, Taipale J, Uusitalo H. Comparing glaucoma medications and general demographics in a sample of glaucoma patients treated in private practice with nationwide registry data in Finland. Acta Ophthalmol (Copenh). 2020;98: 449–454. doi:10.1111/aos.14338

4. Parkkari M, Purola P, Uusitalo H. Ocular surface disease signs and symptoms of glaucoma patients and their relation to glaucoma medication in Finland. Eur J Ophthalmol. 2023;33: 993–1002. doi:10.1177/11206721221144339

5. Schwartz GF, Quigley HA. Adherence and Persistence with Glaucoma Therapy. Surv Ophthalmol. 2008;53: S57–S68. doi:10.1016/j.survophthal.2008.08.002

6. Yeaw J, Benner JS, Walt JG, Sian S, Smith DB. Comparing Adherence and Persistence Across 6 Chronic Medication Classes. J Manag Care Pharm. 2009;15: 728–740. doi:10.18553/jmcp.2009.15.9.728

7. Reardon G, Kotak S, Schwartz GF. Objective assessment of compliance and persistence among patients treated for glaucoma and ocular hypertension: a systematic review. Patient Prefer Adherence. 2011;5: 441–463. doi:10.2147/PPA.S23780

8. Sleath B, Blalock S, Covert D, Stone JL, Skinner AC, Muir K, et al. The Relationship between Glaucoma Medication Adherence, Eye Drop Technique, and Visual Field Defect Severity. Ophthalmology. 2011;118: 2398–2402. doi:10.1016/j.ophtha.2011.05.013

9. Diestelhorst M, Schaefer CP, Beusterien KM, Plante KM, Fain JM, Mozaffari E, et al. Persistency and Clinical Outcomes Associated with Latanoprost and Beta-Blocker Monotherapy: Evidence from a European Retrospective Cohort Study. Eur J Ophthalmol. 2003;13: 21–29. doi:10.1177/112067210301304S03

10. Newman-Casey PA, Blachley T, Lee PP, Heisler M, Farris KB, Stein JD. Patterns of Glaucoma Medication Adherence over Four Years of Follow-Up. Ophthalmology. 2015;122: 2010–2021. doi:10.1016/j.ophtha.2015.06.039

11. Goldshtein I, Shalev V, Zigman N, Chodick G, Levkovitch-Verbin H. The Maccabi Glaucoma Study: Treatment Patterns and Persistence With Glaucoma Therapy in a Large Israeli Health Maintenance Organization. J Glaucoma. 2016;25: e386-391. doi:10.1097/IJG.0000000000000380

12. Kashiwagi K, Chono E, Koesters S, Yap PS. Persistence and treatment patterns of fixed combination drugs for glaucoma: a retrospective claims database study in Japan. BMC Ophthalmol. 2020;20: 223. doi:10.1186/s12886-020-01508-8

13. Schwartz GF, Patel A, Naik R, Lunacsek O, Ogbonnaya A, Campbell J. Characteristics and Treatment Patterns of Newly Diagnosed Open-Angle Glaucoma Patients in the United States: An Administrative Database Analysis. Ophthalmol Glaucoma. 2021;4: 117–125. doi:10.1016/j.ogla.2020.09.002

14. McClelland JF, Bodle L, Little J-A. Investigation of medication adherence and reasons for poor adherence in patients on long-term glaucoma treatment regimes. Patient Prefer Adherence. 2019;13: 431–439. doi:10.2147/PPA.S176412

15. Koponen P, Borodulin K, Lundqvist A, Sääksjärvi K, Koskinen S. Health, functional capacity and welfare in Finland – FinHealth 2017 study (in Finnish). Helsinki: National Institute for Health and Welfare (THL), Report 4/2018; 2018. Available: https://www.julkari.fi/handle/10024/136223

16. Borodulin K, Sääksjärvi K. FinHealth 2017 Study – Methods. Helsinki: Finnish Institute for Health and Welfare (THL), Report 17/2019; 2019. Available: https://www.julkari.fi/handle/10024/139084

17. Lumley T. Analysis of Complex Survey Samples. J Stat Softw. 2004;009. Available: https://ideas.repec.org/a/jss/jstsof/v009i08.html

18. Therneau TM, Grambsch PM. Modeling survival data: extending the Cox model. New York: Springer; 2000.

19. Therneau T. A Package for Survival Analysis in R. R package version 3.5-8. 2024. Available: https://CRAN.R-project.org/package=survival

20. Bogaart P. Package ‘PantaRhei.’ 2020 [cited 12 Jan 2024]. Available: https://cran.r-project.org/web/packages/PantaRhei/vignettes/panta-rhei.html

21. Signorell A, Aho K, Alfons A, Anderegg N, Aragon T, Arppe A, et al. DescTools: Tools for descriptive statistics. R Package Version 099. 2019;28: 17.

22. Newman AR, Andrew NH. Changes in Australian practice patterns for glaucoma management. Clin Experiment Ophthalmol. 2019;47: 571–580. doi:10.1111/ceo.13456

23. Kim CY, Park KH, Ahn J, Ahn M-D, Cha SC, Kim HS, et al. Treatment patterns and medication adherence of patients with glaucoma in South Korea. Br J Ophthalmol. 2017;101: 801–807. doi:10.1136/bjophthalmol-2016-308505

24. Schwartz GF, Reardon G, Mozaffari E. Persistency with latanoprost or timolol in primary open-angle glaucoma suspects. Am J Ophthalmol. 2004;137: S13–S16. doi:10.1016/j.ajo.2003.10.034

25. Nordstrom BL, Friedman DS, Mozaffari E, Quigley HA, Walker AM. Persistence and Adherence With Topical Glaucoma Therapy. Am J Ophthalmol. 2005;140: 598.e1-598.e11. doi:10.1016/j.ajo.2005.04.051

26. Hwang D-K, Liu CJ-L, Pu C-Y, Chou Y-J, Chou P. Persistence of Topical Glaucoma Medication: A Nationwide Population-Based Cohort Study in Taiwan. JAMA Ophthalmol. 2014;132: 1446–1452. doi:10.1001/jamaophthalmol.2014.3333

27. Jang Y, Jee D, Lee D, Choi N-K, Bae S. Medication Adherence and Persistence of Open-Angle Glaucoma Patients in Korea: A Retrospective Study Using National Health Insurance Claims Data. Int J Environ Res Public Health. 2021;18: 4106. doi:10.3390/ijerph18084106

28. Tuulonen A, Airaksinen PJ, Erola E, Forsman E, Friberg K, Kaila M, et al. The Finnish evidence-based guideline for open-angle glaucoma. Acta Ophthalmol Scand. 2003;81: 3–18.

29. Gazzard G, Konstantakopoulou E, Garway-Heath D, Garg A, Vickerstaff V, Hunter R, et al. Selective laser trabeculoplasty versus eye drops for first-line treatment of ocular hypertension and glaucoma (LiGHT): a multicentre randomised controlled trial. The Lancet. 2019;393: 1505–1516. doi:10.1016/S0140-6736(18)32213-X

Figure1 Click here to access/download;Figure;Fig1.tif

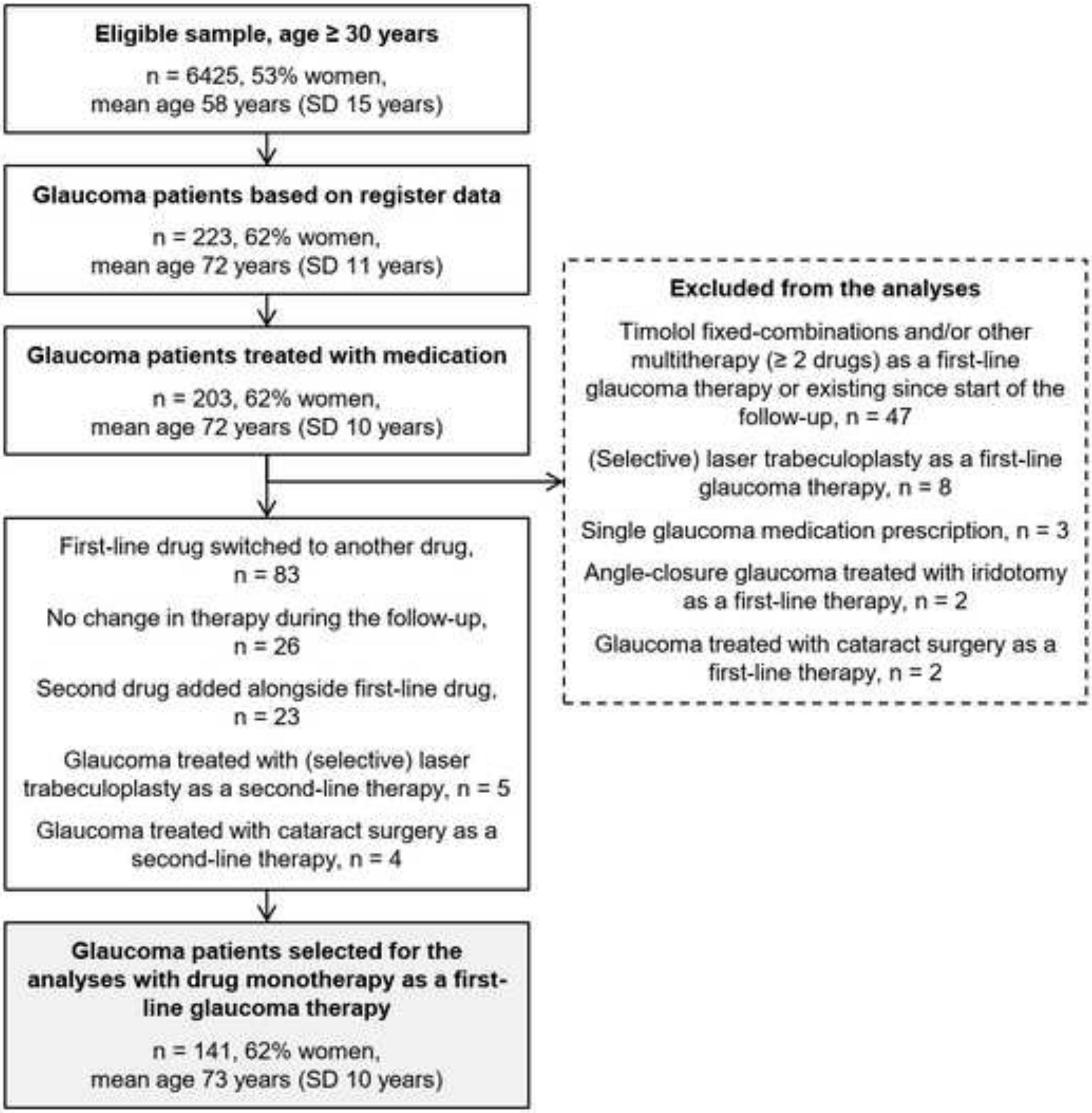

Figure2

Click here to access/download;Figure;Fig2.tif

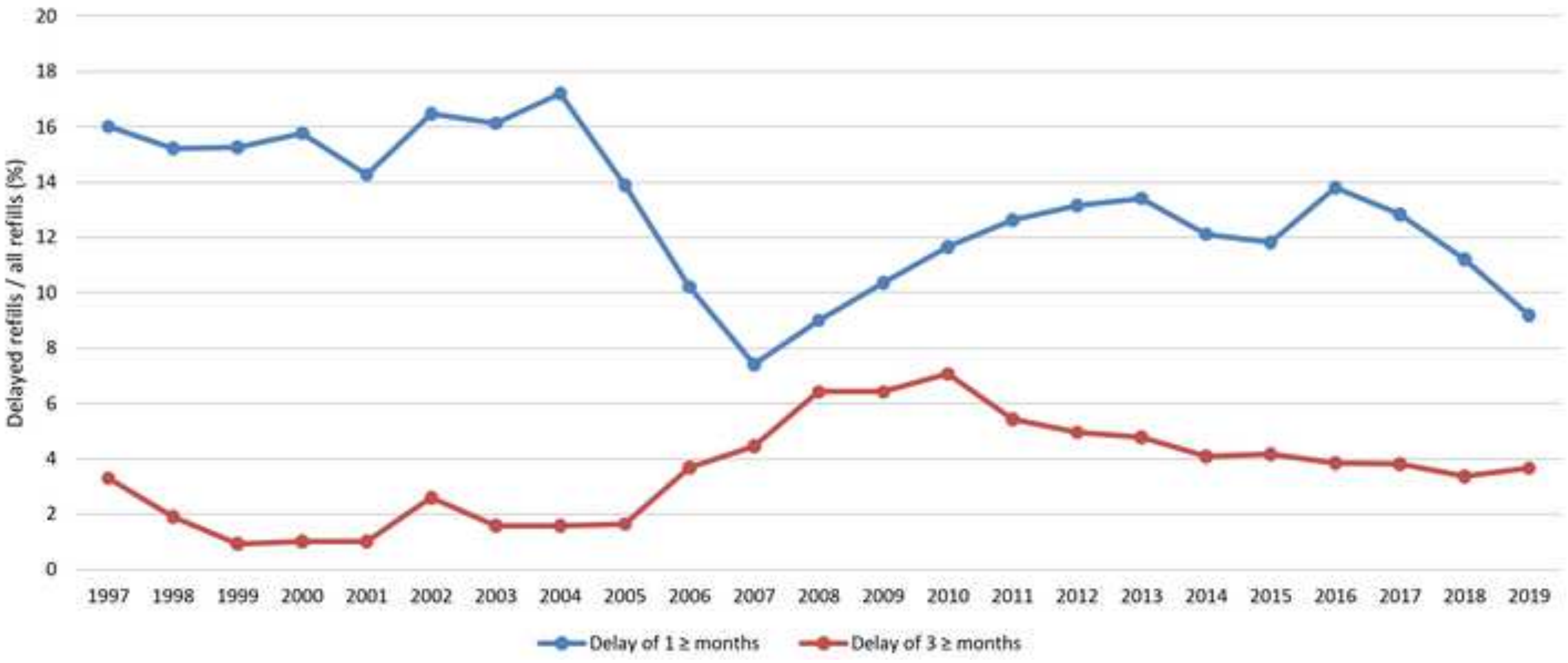

Figure3

Click here to access/download;Figure;Fig3.tif

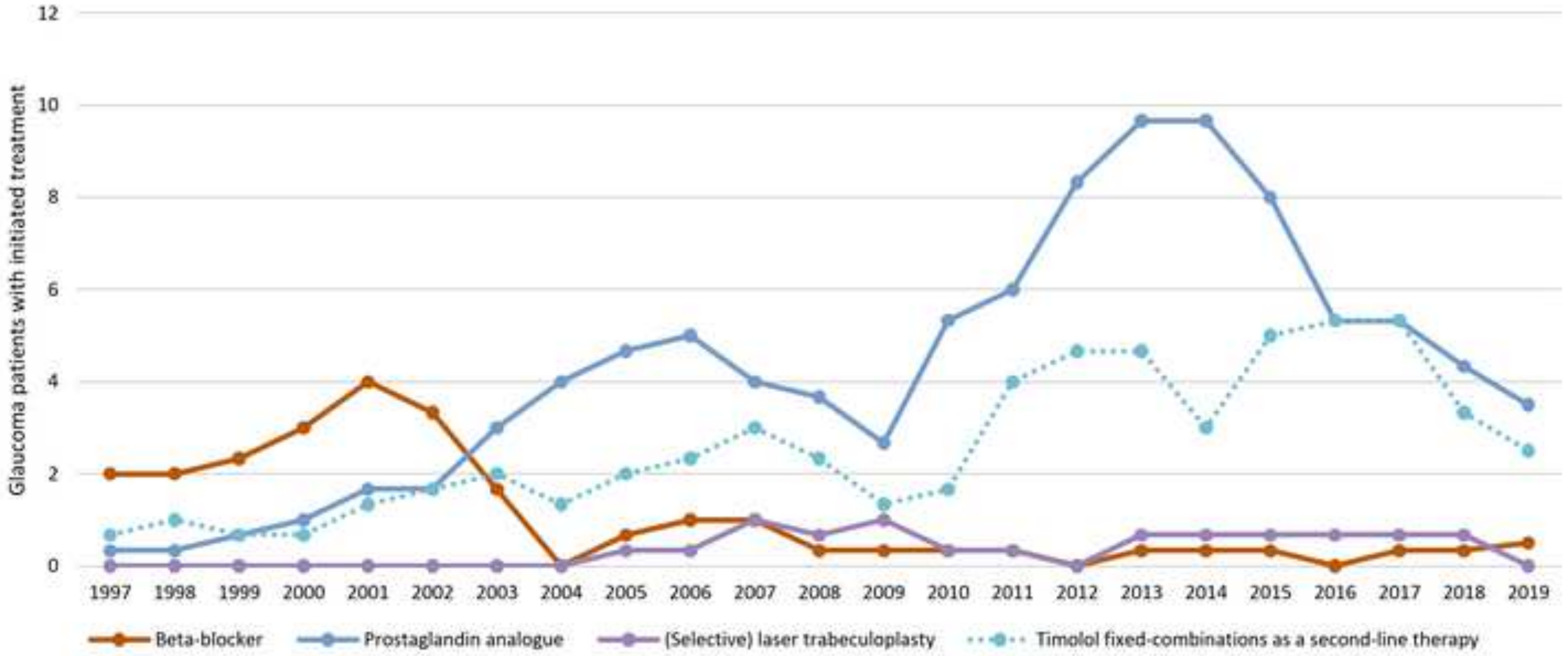

Figure4

Click here to access/download;Figure;Fig4.tif

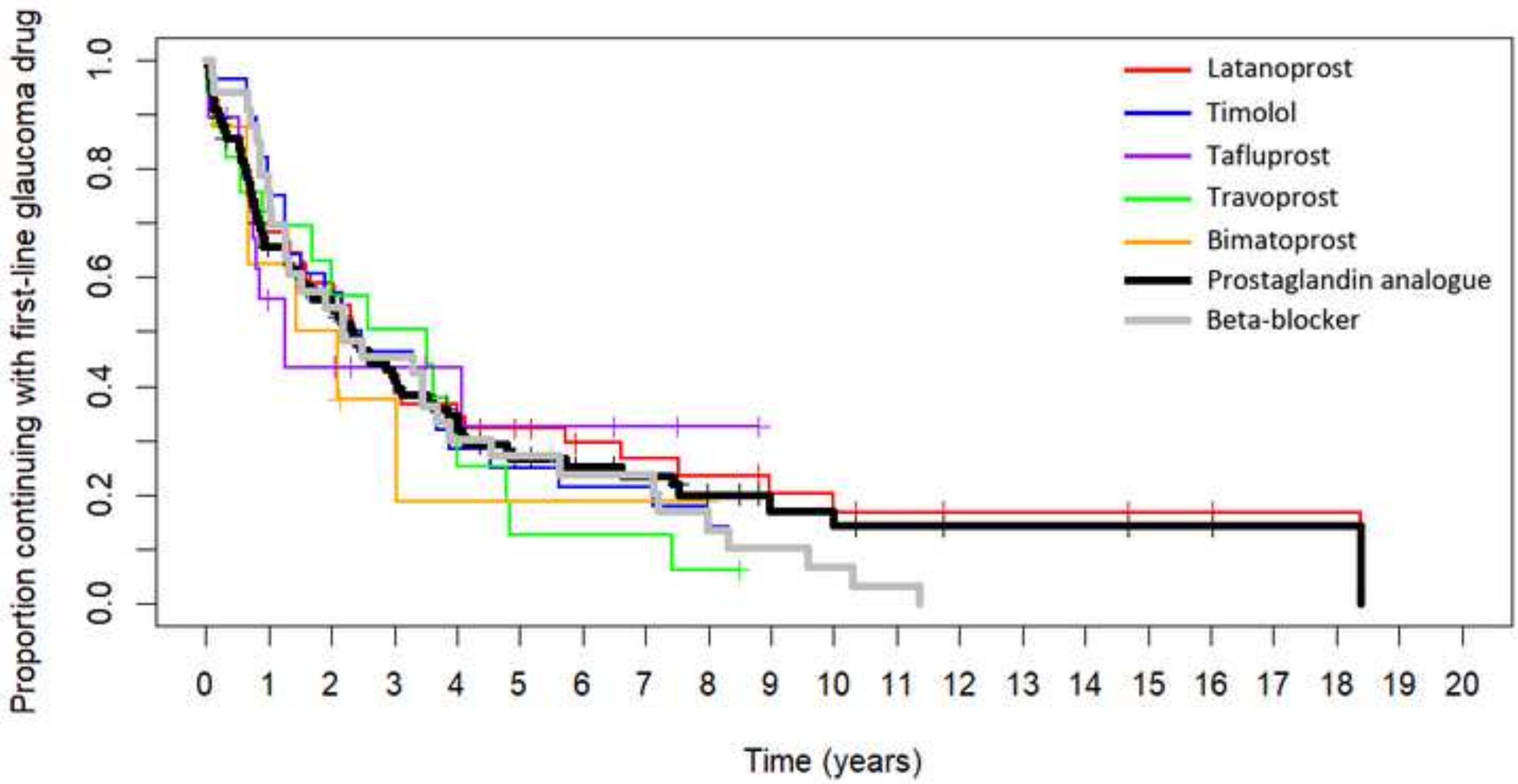

Figure5 Click here to access/download;Figure;Fig5.tif

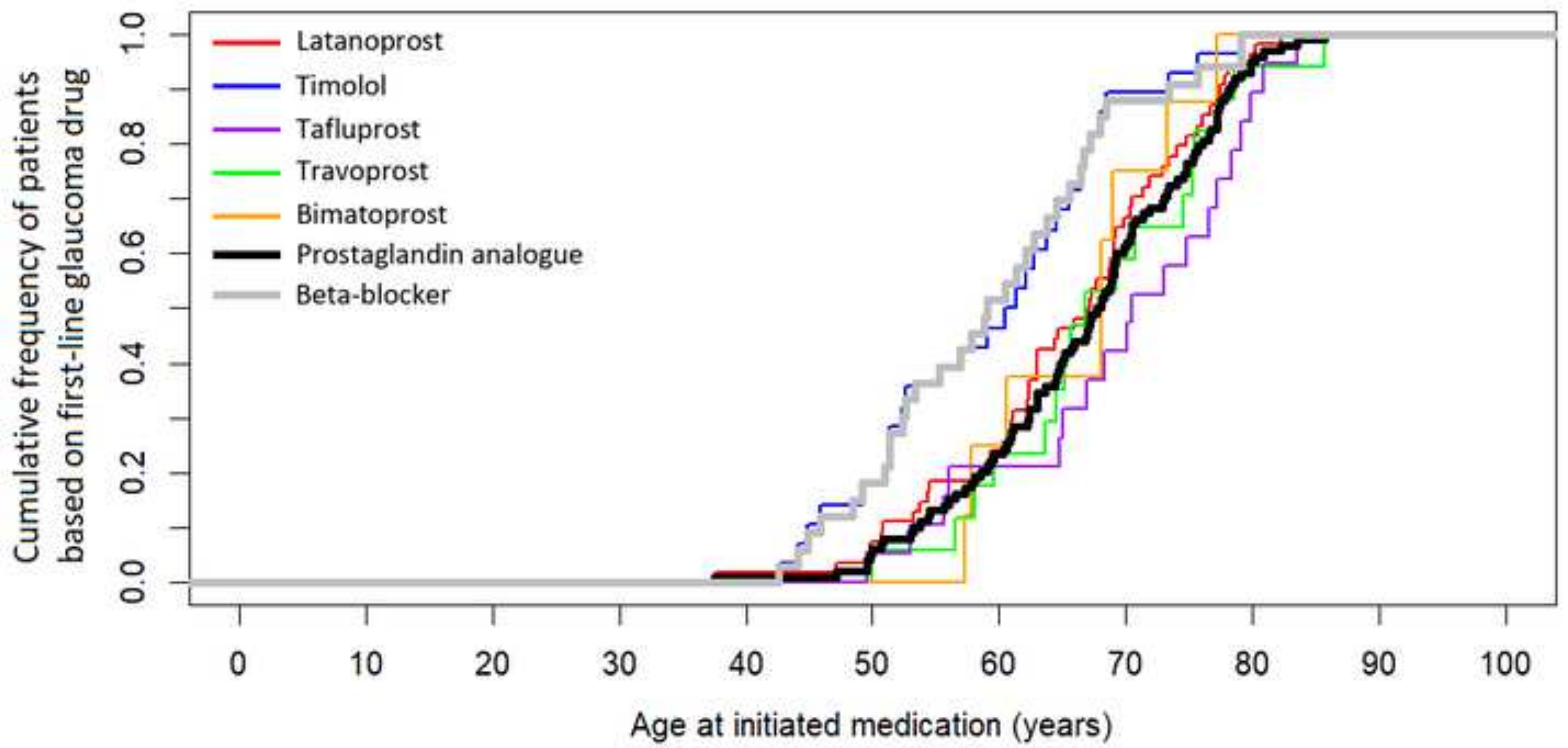

Figure6

Click here to access/download;Figure;Fig6.tif

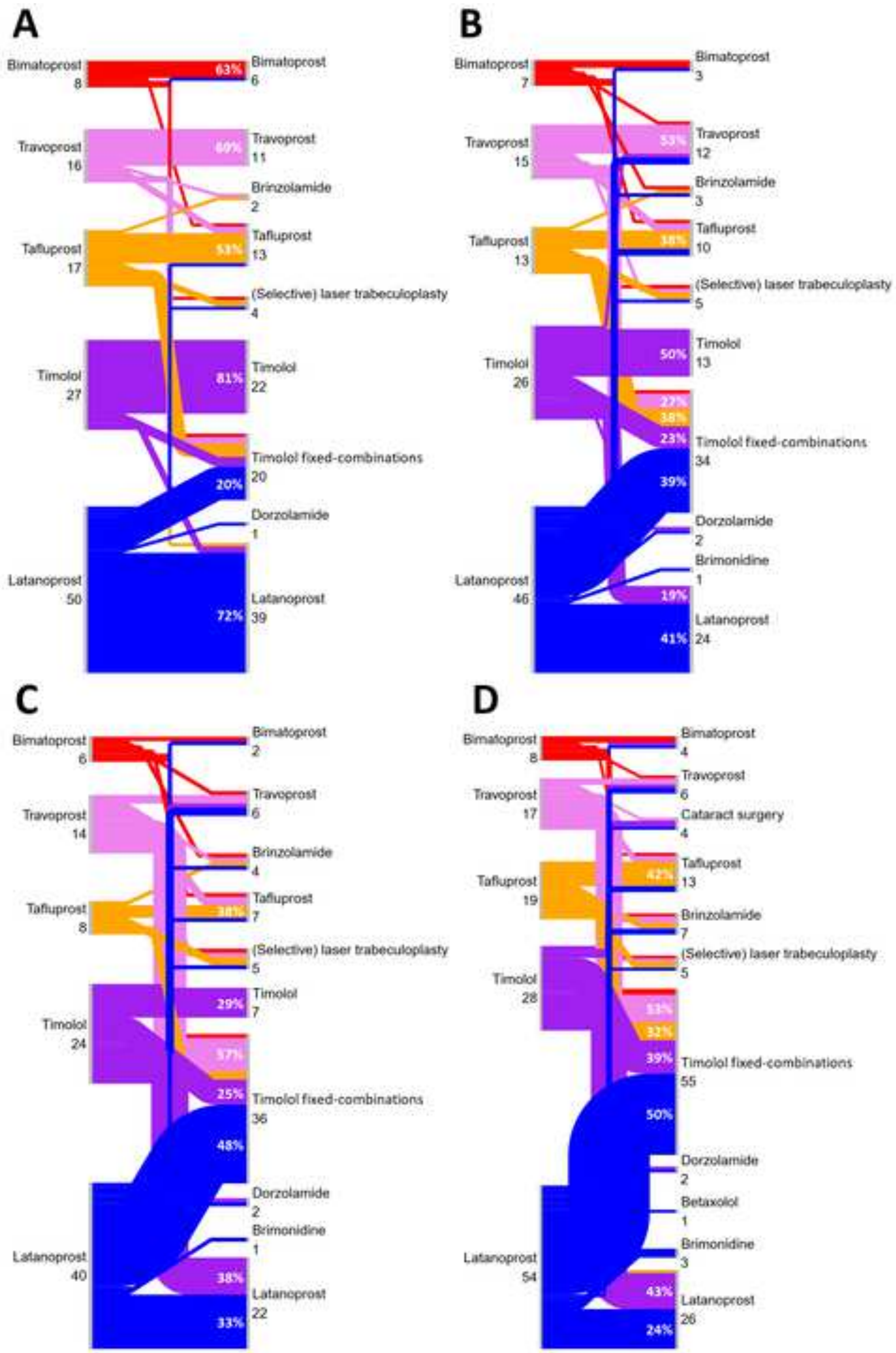

Supplement: Attachment — Submitted filename: PONE-D-24-25991_reviewed.pdf [file pone.0316835.s002.pdf]
