# Supplementary material for: First-line glaucoma monotherapy medication patterns in Finland during 1995–2019 based on a population-based study
Source: PLoS One. 2025 Jan 30;20(1):e0316835. doi: 10.1371/journal.pone.0316835 (PMC11781729; doi:10.1371/journal.pone.0316835)
Supplement: Attachment — Submitted filename: renamed_88eb4.docx [file pone.0316835.s003.docx]

**General comments**

This research makes a significant contribution to ongoing work in medication adherence and provides a comprehensive account of changes in the use of prescribed glaucoma therapies over 25 years. The paper could be strengthened by including some discussion about the reasons for some of the changes that were observed, as well as possible clinical implications of these findings.

**Minor comments**

1. Abstract line 25: Consider stating “drawn from” instead of “drawn for”
2. Abstract line 36: Consider using “significantly longer” or “longer” instead of “statistically significantly longer” since any definitive statements should already be statistically significant
3. Abstract line 45: Consider stating “remain clinical challenges” instead of “This decline, together with the consistent problem of non-persistence remain a clinical challenge” Use plural form as there are two subjects (the decline, problem of non-persistence)
4. Manuscript Line 47: Consider using “group” instead of “collection”
5. Manuscript Line 52: Consider stating “ocular hypertensives” (plural)
6. Manuscript Line 54: Consider stating “in case the current” instead of “in the case the current” (“the” is not needed)
7. Manuscript Line 83: Consider stating “To summarize, the sample was drawn using two-stage stratified cluster sampling” (Replace “by a”)
8. Manuscript Line 88: Consider stating “For the present study we selected 9,288 participants” (Remove “the”)
9. Manuscript Line 126: Consider stating “and a weighting scheme” (Add ‘a”)
10. Consider moving the “Ethics approval and informed consent” section to after the Study design section
11. Manuscript line 192: Please delete “in specific”. It is not needed for the reader to understand the context and its placement is a bit odd. If the authors would like to keep, please consider using “specifically” instead.
12. Manuscript Line 249: Please consider rephrasing to “determined that timolol, with a mean of 59.6 years (95% CI 56.0–63.1) was associated with the lowest age…”
13. Manuscript Line 292: Consider stating “the ageing population”
14. Figure 3: Please include a unit for the y-axis. I believe this should be “%”

**Major comments**

1. If possible, the authors should provide a demographic summary of the participants whose data are analyzed in the paper, particularly given that authors’ statement about previous research not being representative. The authors should also specify the type/types of glaucoma, as the treatment approach is not the same (primary vs secondary, open vs closed angle)
2. Forgive me if I missed this, but why are the authors only interested in monotherapy? Has this been stated in the manuscript? Are there any plans to analyze patterns in polytherapy?
3. Table 1: Consider omitting the “%” symbol from the right column since it is already included in the column heading. Consider making this change for all tables
4. The authors state that “During the 25-year follow-up the proportion of delays of 1 ≥ months decreased while the proportion of delays of 3 ≥ months increased.” It would be nice for the authors to share some insights about possible reasons for this
5. The authors state that “However, after 5 years of follow-up the length of use was statistically significantly longer for prostaglandin analogues compared with beta-blockers.” Similar comment here. These are interesting results, but readers of the paper would be interested in potential reasons. Perhaps differences in side effect profiles, efficacy etc?
6. The authors state that “In our study, approximately 21% of glaucoma patients treated with medication had their therapy initiated with multiple antiglaucoma agents. This figure is in line with a population-based study conducted in Israel in 2003–2012, in which 22% of glaucoma patients treated with medication had either a combination drug or several separate drugs with multitherapy as a first-line glaucoma therapy [11]. This behavior is contradictory to the glaucoma guideline [28], and the reason for it is not known.” I believe that more is needed here. The authors call out this apparent contradiction. Naturally, readers will want to know more about possible explanations or even clinical implications of this finding.
7. Do the authors have other plans to explore patterns of medication use? For instance, the Newman-Casey paper identified distinct patterns using pharmacy claims/refill data: (1) never adherent after their index prescription fill (7.5% and 15.6% of persons in the 1- and 4-year models, respectively); (2) persistently very poor adherence (14.9% and 23.4% of persons in the 1- and 4-year models, respectively); (3) declining adherence (9.5% and 9.1% of persons in the 1- and 4-year models, respectively); (4) persistently moderate adherence (48.1% and 37.0% of persons in the 1- and 4-year models, respectively); and (5) persistently good adherence (20.0% and 15.0% of persons in the 1- and 4-year models, respectively). With up to 25 years of data, this analysis could be very impactful and informative since Newman-Casey’s findings were observed over only 4 years.
