# Supplementary material for: First-line glaucoma monotherapy medication patterns in Finland during 1995–2019 based on a population-based study
Source: PLoS One. 2025 Jan 30;20(1):e0316835. doi: 10.1371/journal.pone.0316835 (PMC11781729; doi:10.1371/journal.pone.0316835)
Supplement: Attachment — Submitted filename: Response to Reviewers.docx [file pone.0316835.s004.docx]

Dear Editor,

We would like to thank you and the reviewers for the valuable comments. We have carefully analyzed all the comments and responded accordingly. Please find below the detailed list of the changes we have made and the answers and comments to the reviewers’ remarks.

**Revisions for Editor:**

*1. Please ensure that your manuscript meets PLOS ONE's style requirements, including those for file naming. The PLOS ONE style templates can be found at https://journals.plos.org/plosone/s/file?id=wjVg/PLOSOne_formatting_sample_main_body.pdf and https://journals.plos.org/plosone/s/file?id=ba62/PLOSOne_formatting_sample_title_authors_affiliations.pdf*

We have rechecked the style requirements as suggested.

*2. We note that you have indicated that there are restrictions to data sharing for this study. For studies involving human research participant data or other sensitive data, we encourage authors to share de-identified or anonymized data. However, when data cannot be publicly shared for ethical reasons, we allow authors to make their data sets available upon request. For information on unacceptable data access restrictions, please see http://journals.plos.org/plosone/s/data-availability#loc-unacceptable-data-access-restrictions.*

*Before we proceed with your manuscript, please address the following prompts:*

*a) If there are ethical or legal restrictions on sharing a de-identified data set, please explain them in detail (e.g., data contain potentially identifying or sensitive patient information, data are owned by a third-party organization, etc.) and who has imposed them (e.g., a Research Ethics Committee or Institutional Review Board, etc.). Please also provide contact information for a data access committee, ethics committee, or other institutional body to which data requests may be sent.*

*b) If there are no restrictions, please upload the minimal anonymized data set necessary to replicate your study findings to a stable, public repository and provide us with the relevant URLs, DOIs, or accession numbers. Please see http://www.bmj.com/content/340/bmj.c181.long for guidelines on how to de-identify and prepare clinical data for publication. For a list of recommended repositories, please see https://journals.plos.org/plosone/s/recommended-repositories. You also have the option of uploading the data as Supporting Information files, but we would recommend depositing data directly to a data repository if possible.*

*Please update your Data Availability statement in the submission form accordingly.*

We have updated the Data Availability statement accordingly:

“The data from FinHealth 2017 and its precursors Health 2000 and Health 2011 are not publicly available as they include confidential information that could compromise the privacy of the participants. The data can be used for research and monitoring of health, wellbeing, functioning, and use of services of the population at THL and with collaborators based on collaboration agreement (more information: terveys-2000-2011@thl.fi). The data available from the THL Biobank cover those who have participated in the health examination and have given consent to biobanking and can be applied via the THL Biobank in accordance with the biobank act and THL Biobank research areas (thl.fi/biobank). The Finnish Social and Health Data Permit Authority Findata may grant permits in accordance with the act on the secondary use of social and health data in Finland (www.findata.fi/en). Further inquiries can be directed to the email address terveys-2000-2011@thl.fi.”

*3. Please include your full ethics statement in the ‘Methods’ section of your manuscript file. In your statement, please include the full name of the IRB or ethics committee who approved or waived your study, as well as whether or not you obtained informed written or verbal consent. If consent was waived for your study, please include this information in your statement as well.*

We have now included the full ethics statement as suggested: “The FinHealth 2017 Survey was approved by the Coordinating Ethics Committee at the Hospital District of Helsinki and Uusimaa with reference 37/13/02/00/2016. All participants received an information letter regarding the study beforehand. Written informed consent was obtained from all participants.”

*4. Please review your reference list to ensure that it is complete and correct. If you have cited papers that have been retracted, please include the rationale for doing so in the manuscript text, or remove these references and replace them with relevant current references. Any changes to the reference list should be mentioned in the rebuttal letter that accompanies your revised manuscript. If you need to cite a retracted article, indicate the article’s retracted status in the References list and also include a citation and full reference for the retraction notice.*

We have reviewed the reference list as suggested. We added three new references as suggested by Reviewer #1:

Quaranta, L., Novella, A., Tettamanti, M., Pasina, L., Weinreb, R. N., & Nobili, A. (2023). Adherence and persistence to medical therapy in glaucoma: An overview. Ophthalmology and therapy, 12(5), 2227-2240.

Tse, A. P., Shah, M., Jamal, N., & Shaikh, A. (2016). Glaucoma treatment adherence at a United Kingdom general practice. Eye, 30(8), 1118-1122.

Menino, J., Camacho, P., & Coelho, A. (2024). Persistence with medical glaucoma therapy in newly diagnosed patients. Medical Hypothesis, Discovery and Innovation in Ophthalmology, 13(2), 63.

**Revisions for Reviewer #1:**

*1. The subheadings of various sections in the article are of similar boldness and size. I recommend redistributing them to facilitate readers' immediate understanding of the overall structure of the article.*

We have now italicized the secondary headings for better clarity as suggested.

*2. While the references provided by the authors are indeed well-targeted and authoritative, and they appear in their appropriate locations, it is worth noting that many of them are over 5 years old, with some reaching 10 years or even 20 years. In particular, references 5-8, which aim to showcase the research on medication adherence among glaucoma patients and highlight the importance of adherence in disease control, are review articles from 13-16 years ago. If possible, replacing these with more recent literature could enhance the persuasiveness of the article's arguments.*

We thank the Reviewer for the suggestion. We have now added the following references:

Quaranta, L., Novella, A., Tettamanti, M., Pasina, L., Weinreb, R. N., & Nobili, A. (2023). Adherence and persistence to medical therapy in glaucoma: An overview. Ophthalmology and therapy, 12(5), 2227-2240.

Tse, A. P., Shah, M., Jamal, N., & Shaikh, A. (2016). Glaucoma treatment adherence at a United Kingdom general practice. Eye, 30(8), 1118-1122.

Menino, J., Camacho, P., & Coelho, A. (2024). Persistence with medical glaucoma therapy in newly diagnosed patients. Medical Hypothesis, Discovery and Innovation in Ophthalmology, 13(2), 63.

*3. In the discussion section, a deeper exploration of the potential factors leading to changes in medication usage patterns could be beneficial, such as patients' subjective experiences, changes in healthcare policies, and the impact of patient education. The article frequently mentions that prostaglandins rapidly replaced beta-blockers; however, we know that prostaglandins are typically administered once daily. Could you further analyze whether the increased adherence is due to the reduced frequency of medication or fewer adverse effects associated with the drugs? I believe such analysis and outlook would provide meaningful guidance for clinical practice.*

The reviewer is right, and we believe such analysis could be important. Unfortunately our data do not allow to distinguish between reduced frequency of medication or fewer adverse effects associated with the drugs, as we only know that the medication has been bought based on the prescription data.

*4. In line 340, you mention that the first-line medication situation in Finland differs from that in Israel. As this article is set to be published in a global open-access journal, it would be beneficial to provide an explanation for this difference to enhance its international relevance. A similar situation arises in line 308 regarding the potential reasons for better medication adherence among glaucoma patients in the United States. Addressing these points would significantly improve the article.*

The Reviewer rises an important point. We have now added the following sentence: “These differences could be explained by variations in populations as well as local factors such as availability of drugs, practices in reimbursements, and guidelines.“

**Revisions for Reviewer #2:**

*In the introduction, there is a need for the disease "glaucoma" to be well defined.*

We thank the Reviewer for the suggestion. We have carefully re-read the manuscript and we believe that the first paragraph of the Introduction will give a sufficient description of glaucoma.

*Minor grammatical edits is required and highlighted.*

We thank the reviewer for pointing out these errors, and we have corrected them as suggested.

*This is the abbreviation for Light Amplification by Stimulated Emission of Radiation. The abbreviation is better written in captial letters.*

In most glaucoma literature, laser is written lowercase, and as such we have left it as it is. For example, see the EGS glaucoma guidelines (ISBN 978-88-98320-39-4).

*“3 had only a single glaucoma medication prescription during the follow-up” Why were these 3 excluded since they had monotherapy? Was monotherapy instituted on diagnosis (baseline)? Does a single glaucoma medication mean the drug was applied to only one eye? Please clarify.*

The 3 subjects were excluded as they only had a single glaucoma medication prescription during the follow-up, indicating that they did not have a continuous medical treatment of glaucoma and did either have other condition than glaucoma or other type of therapy.

*Results were clearly presented. However, there are a lot of repetition of results in the discussion.*

We thank the Reviewer for the suggestion. We have now simplified the results in the Discussion, e.g., by omitting the repetition of the percentages.

**Revisions for Reviewer #3:**

*This is a very interesting study created with an amount of longitudinal linked data that is rarely available. Nonetheless, the small number of patients included in the analysis is something to be considered (n=141).*

We thank the Reviewer for rising an important point. The small number of patients is due to the nature of population-based studies aiming to have a nationally representative sample. In this study the original sample size of the survey was 10247 Finnish adults aged 18 years or older which should regarded as a large one. We have taken the sample size into account by utilizing population weights and calculating confidence intervals.

*Lines 55 to 57 – laser is not only a first line therapy for those specific cases. It has a much broader indication as first-line therapy*

Thank you for the comment. We have now revised the sentence: “Alternatively, laser treatments such as laser trabeculoplasty and laser iridotomy or surgery can be offered as a first-line glaucoma therapy, in patients with drug-related adverse reactions, in patients with difficulties in administering topical medication, and in patients with severe disease.”

*Results – would mention the prevalence of glaucoma patients (as per this methodology) in the population sample - +- 2.2% which matches other studies from European countries. (eg* [*https://pubmed.ncbi.nlm.nih.gov/28368997/*](https://pubmed.ncbi.nlm.nih.gov/28368997/)*)*

The prevalence of register-based glaucoma was 3.1%, and the prevalence of glaucoma treated with first-line drug monotherapy 2.0% in the population 30 years or older. We have added these figures to the Results, first paragraph.

*Figure 3 – what is the unit of the y axis?*

The unit of the y axis is the mean n of a 3-year central moving average, which we have now added to the figure.

*Figure 3 – what do you believe is the reason for the PGA trend shape? Increasing until 2014 and then decreasing?*

Considering that the first Finnish Glaucoma Guidelines was published in 1999 with consequently increased awareness of the disease, it is likely that there was a surge of newly diagnosed glaucoma patients at the time and a subsequent decrease in new diagnoses. We this believe could explain the decreasing trend of the PGA after 2014 considering the decreasing number of new glaucoma cases in the FinHealth 2017 survey sample as seen in the example figure below.

Figure. Dates of first glaucoma-related diagnoses and special reimbursements among the 141 analyzed glaucoma patients with initiated monotherapy

n

|  | Date of first special reimbursement for glaucoma medication | Date of first glaucoma diagnosis |
| --- | --- | --- |
| 1995 | 4 |  |
| 1996 | 2 |  |
| 1997 | 1 |  |
| 1998 | 3 | 4 |
| 1999 | 2 | 1 |
| 2000 | 2 | 2 |
| 2001 | 6 | 1 |
| 2002 | 6 | 3 |
| 2003 | 2 | 2 |
| 2004 | 8 | 5 |
| 2005 | 5 | 3 |
| 2006 | 5 | 5 |
| 2007 | 8 | 5 |
| 2008 | 2 | 4 |
| 2009 | 1 | 4 |
| 2010 | 6 | 7 |
| 2011 | 8 | 9 |
| 2012 | 4 | 3 |
| 2013 | 13 | 3 |
| 2014 | 14 | 6 |
| 2015 | 8 | 9 |
| 2016 | 5 | 5 |
| 2017 | 6 | 1 |
| 2018 | 3 |  |
| 2019 | 6 |  |

*Would maybe say something at the end of results regarding the frequency of switching to another monotherapy PGA versus switching to a fixed combination with timolol.*

The frequency of switching has been presented in Figure 6. We have now added the switching frequencies:

”During 1 year of follow-up, 9% switched to another monotherapy, 17% switched to Timolol fixed-combinations, and 3% switched to (selective) laser trabeculoplasty. During 3 years of follow-up, 20% switched to another monotherapy, 32% switched to Timolol fixed-combinations, and 5% switched to (selective) laser trabeculoplasty. During 5 years of follow-up, 27% switched to another monotherapy, 39% switched to Timolol fixed-combinations, and 5% switched to (selective) laser trabeculoplasty. And during the total follow-up, 29% switched to another monotherapy, 44% switched to Timolol fixed-combinations, and 4% switched to (selective) laser trabeculoplasty.”

Discussion

*Lines 307 to 315 – could the amount of PXF glaucoma in Finland (versus almost anywhere else) be a factor in the need to move from the initial therapy sooner?*

We agree the with the suggestion of the Reviewer, and we have now added the higher prevalence of exfoliative glaucoma being a possible explanation: “In addition, the high prevalence of exfoliative glaucoma in Finland could be a factor in the need to move from the initial therapy sooner.”

*Would it make sense to discuss the possibility for tachyphylaxis with betablockers?*

This would be interesting, but our data are not allowing us for detailed discussion. In addition to decreasing efficacy of betablocker treatment, the reason for withdrawal of betablockers can be, for example, systemic or local side-effects. Unfortunately our data are not allowing to differentiate between them.

*I understand that the lower mean age with the beta blockers may indeed be biased, but could this also be because older patients may have more pathology that contra indicates betablockers and also the avoidance of PGA in young people since they can lead to PGA-related orbitopathy?*

We thank the Reviewer for rising an interesting point. However, the changes in the availability of various glaucoma drugs during the follow-up period are overriding these possible explanations and were therefore very difficult to study.

**Revisions for Reviewer #4:**

*Very interesting research. Please expand on the possible reasons/implications of these findings in order to appeal to clinicians/a wider audience who may be interested in policy changes or intervention planning*

We thank the Reviewer for the comment. We have added the following points to the second paragraph in Discussion: “The results of this study demonstrate the individual nature of glaucoma and indicate the necessity of careful follow-up of the glaucoma patients, individual tailoring of glaucoma therapy, and broader use of therapies like selective laser trabeculoplasty, however, not forgetting the individual efficacy of them.”

**Revisions for Reviewer #5:**

*• In the methods section: "The authors chose non-parametric tests (Mann–Whitney U and Kruskal–Wallis) due to data skewness. For added clarity, the authors might specify which variables were analyzed with each test, as this context would strengthen the rationale behind the analysis choices.*

We have rephrased the sentence: “Therefore, Mann–Whitney U test was used for analyzing the mean age at initiation of therapy of the two most common first-line glaucoma drug classes and Kruskal–Wallis test for the analysis of the five most common drug types adjusted with Dunn–Bonferroni correction --”.

*• In the discussion: the authors reference the same study from Israel (2003–2012) multiple times, which may create the impression of separate studies. Combining all comparisons with this study into a single paragraph would improve clarity and coherence.*

The Reviewer is quite right. However, we have been referring the various aspects of the article of Goldshtein et al. In different paragraphs of the Discussion. To keep the structure of the Discussion consistent, we found it difficult to follow the Reviewer’s suggestion.

*• In the discussion: While the conclusion is strong, the authors could further highlight the broader implications of the findings and suggest practical pathways for enhancing glaucoma care. They might consider adding a brief note on future research directions, such as exploring factors influencing non-persistence, assessing the impact of newer treatment options on long-term outcomes, or evaluating patient education strategies to improve adherence*

We have added the following points to the second paragraph in Discussion: “The results of this study demonstrate the individual nature of glaucoma and indicate the necessity of careful follow-up of the glaucoma patients, individual tailoring of glaucoma therapy, and broader use of therapies like selective laser trabeculoplasty, however, not forgetting the individual efficacy of them.”

*• In the discussion: The limitations are valid. To further strengthen this section, consider noting the potential impact of excluding patients who started on combination therapy, as this may affect the representativeness of the findings, making them most applicable to patients initiating monotherapy.*

We have now added this to the limitations of the study as suggested: “We excluded patients who started on combination therapy, which may affect the representativeness of the findings and make them most applicable to patients initiating monotherapy.”

*• In the discussion: The study reveals a significant early decline in the continuation of first-line monotherapy, as well as a shift from beta-blockers to prostaglandin analogues as the preferred first-line therapy. Notably, prostaglandin analogues showed greater persistence beyond five years compared to beta-blockers. Expanding the discussion to explore possible reasons for both the early decline and this therapeutic shift—such as factors affecting adherence, improved intraocular pressure (IOP) control, convenience, or fewer side effects—could provide deeper insights into the practical implications for glaucoma management.*

On the basis of our data, it is unfortunately not possible to make such conclusions. Factors such as adherence, improved intraocular pressure (IOP) control, convenience, or fewer side effects may well have been affected these changes, but we can not assess the role of these factors.

*• "The authors could consider revising the text to use active voice, as this adjustment may enhance the clarity and engagement of the writing."*

We have increased the use of active voice throughout the manuscript as suggested.

**Revisions for Reviewer #6:**

Minor comments

*1. Abstract line 25: Consider stating “drawn from” instead of “drawn for”*

*2. Abstract line 36: Consider using “significantly longer” or “longer” instead of “statistically significantly longer” since any definitive statements should already be statistically significant*

*3. Abstract line 45: Consider stating “remain clinical challenges” instead of “This decline, together with the consistent problem of non-persistence remain a clinical challenge” Use plural form as there are two subjects (the decline, problem of non-persistence)*

*4. Manuscript Line 47: Consider using “group” instead of “collection”*

*5. Manuscript Line 52: Consider stating “ocular hypertensives” (plural)*

*6. Manuscript Line 54: Consider stating “in case the current” instead of “in the case the current” (“the” is not needed)*

*7. Manuscript Line 83: Consider stating “To summarize, the sample was drawn using two-stage stratified cluster sampling” (Replace “by a”)*

*8. Manuscript Line 88: Consider stating “For the present study we selected 9,288 participants” (Remove “the”)*

*9. Manuscript Line 126: Consider stating “and a weighting scheme” (Add ‘a”)*

*10. Consider moving the “Ethics approval and informed consent” section to after the Study design section*

*11. Manuscript line 192: Please delete “in specific”. It is not needed for the reader to understand the context and its placement is a bit odd. If the authors would like to keep, please consider using “specifically” instead.*

*12. Manuscript Line 249: Please consider rephrasing to “determined that timolol, with a mean of 59.6 years (95% CI 56.0–63.1) was associated with the lowest age…”*

*13. Manuscript Line 292: Consider stating “the ageing population”*

*14. Figure 3: Please include a unit for the y-axis. I believe this should be “%”*

We have made the corrections as suggested.

Major comments

*1. If possible, the authors should provide a demographic summary of the participants whose data are analyzed in the paper, particularly given that authors’ statement about previous research not being representative. The authors should also specify the type/types of glaucoma, as the treatment approach is not the same (primary vs secondary, open vs closed angle)*

We thank the Reviewer for rising important points. A demographic summary of the participants is provided in Figure 1.

We have now added the different glaucoma diagnoses among the analyzed sample in Table S1:

| **Verified glaucoma diagnosis** | **%** |
| --- | --- |
| Primary/chronic open-angle glaucoma | 41.2 |
| Exfoliative glaucoma | 16.8 |
| Normal-tension glaucoma | 10.1 |
| Unspecified glaucoma | 10.1 |
| Suspected glaucoma | 6.7 |
| Chronic angle-closure glaucoma | 5.0 |
| Glaucoma secondary to other disorder/factor | 5.0 |
| Unspecified open-angle glaucoma | 1.7 |
| Acute angle-closure glaucoma | 1.7 |
| Pigmentary glaucoma | 0.8 |
| Total | n = 119 |

*2. Forgive me if I missed this, but why are the authors only interested in monotherapy? Has this been stated in the manuscript? Are there any plans to analyze patterns in polytherapy?*

We thank the Reviewer for the suggestion for the polytherapy. As the focus of the current manuscript is in monotherapy as stated in the title and other points of the text, we believe analyzing polytherapy deserves a study of its own, which we will consider in the future.

*3. Table 1: Consider omitting the “%” symbol from the right column since it is already included in the column heading. Consider making this change for all tables*

We have removed the %-symbol from the columns in Tables 1 and 2 as suggested. We left it in Table 3 as it is not included in the column headings.

*4. The authors state that “During the 25-year follow-up the proportion of delays of 1 ≥ months decreased while the proportion of delays of 3 ≥ months increased.” It would be nice for the authors to share some insights about possible reasons for this*

We have revised the discussion for this part: “Interestingly, in our study, the proportion of all delayed refills declined during the 25-year follow-up while the proportion of the most severe delays somewhat increased. The general decline in delayed refills could be explained by the increased recognition of the importance of regularly administered medication, whereas the increase in most severe delays likely reflects the ageing of the study cohort, considering the increasing possibility of forgetfulness due to memory disturbances, dementia, and other conditions.”

*5. The authors state that “However, after 5 years of follow-up the length of use was statistically significantly longer for prostaglandin analogues compared with beta-blockers.” Similar comment here. These are interesting results, but readers of the paper would be interested in potential reasons. Perhaps differences in side effect profiles, efficacy etc?*

Based on the valuable comment of the Reviewer, we have added to the Discussion: “This is probably due to the better efficacy of the prostaglandin analogues and the more easy once a day dosing.“

*6. The authors state that “In our study, approximately 21% of glaucoma patients treated with medication had their therapy initiated with multiple antiglaucoma agents. This figure is in line with a population-based study conducted in Israel in 2003–2012, in which 22% of glaucoma patients treated with medication had either a combination drug or several separate drugs with multitherapy as a first-line glaucoma therapy [11]. This behavior is contradictory to the glaucoma guideline [28], and the reason for it is not known.” I believe that more is needed here. The authors call out this apparent contradiction. Naturally, readers will want to know more about possible explanations or even clinical implications of this finding.*

We have revised the end of the statement: “This behavior is contradictory to the glaucoma guideline [28], and the reason for it is not known. If initiated with multitherapy, it is difficult to observe which agents are effective, leading to the possibility of inefficient treatment with increased risk of side-effects. Therefore, it is important to improve the awareness of the guidelines and adherence to them.”

*7. Do the authors have other plans to explore patterns of medication use? For instance, the Newman-Casey paper identified distinct patterns using pharmacy claims/refill data: (1) never adherent after their index prescription fill (7.5% and 15.6% of persons in the 1- and 4-year models, respectively); (2) persistently very poor adherence (14.9% and 23.4% of persons in the 1- and 4-year models, respectively); (3) declining adherence (9.5% and 9.1% of persons in the 1- and 4-year models, respectively); (4) persistently moderate adherence (48.1% and 37.0% of persons in the 1- and 4-year models, respectively); and (5) persistently good adherence (20.0% and 15.0% of persons in the 1- and 4-year models, respectively). With up to 25 years of data, this analysis could be very impactful and informative since Newman-Casey’s findings were observed over only 4 years.*

We thank the Reviewer for the suggestion, and we are seriously considering this in the future studies.

We appreciate the valuable comments of the Editor and reviewers that helped us to improve the manuscript. We hope that the paper could be published in the revised form in your journal.

On behalf of the research group,

Petri Purola, PhD

Tampere University, Department of Ophthalmology

Arvo building, 33014 Tampere University, Finland

E-mail: petri.purola@tuni.fi

Telephone: +358 400 695309
